# Between-group competition and human cooperation

**DOI:** 10.1098/rspb.2008.1060

**Published:** 2008-09-30

**Authors:** Mikael Puurtinen, Tapio Mappes

**Affiliations:** Centre of Excellence in Evolutionary Research, University of JyväskyläPO Box 35, Jyväskylä 40014, Finland

**Keywords:** group competition, cooperation, social dilemma, public goods, morality

## Abstract

A distinctive feature of human behaviour is the widespread occurrence of cooperation among unrelated individuals. Explaining the maintenance of costly within-group cooperation is a challenge because the incentive to free ride on the efforts of other group members is expected to lead to decay of cooperation. However, the costs of cooperation can be diminished or overcome when there is competition at a higher level of organizational hierarchy. Here we show that competition between groups resolves the paradigmatic ‘public goods’ social dilemma and increases within-group cooperation and overall productivity. Further, group competition intensifies the moral emotions of anger and guilt associated with violations of the cooperative norm. The results suggest an important role for group conflict in the evolution of human cooperation and moral emotions.

## 1. Introduction

A distinctive feature of human behaviour is the widespread occurrence of cooperation among unrelated individuals. From an economic and evolutionary viewpoint, the maintenance of such cooperation is puzzling since cooperation is often costly: an individual performing the cooperative act carries the costs of cooperation, while individuals who do not contribute to cooperation gain the benefits without having to pay for the costs. The incentive to free ride on other individuals' efforts when the benefits of cooperation fall upon all members of a group is expected to lead to decay of cooperation, even though members of cooperative groups are better off than members of uncooperative groups.

However, human social interactions are not limited to within-group interactions, but groups also interact and compete. Darwin (1871) proposed that competition between groups could have selected for individual traits such as courage and faithfulness that contribute to a group's success in conflict. Cooperation and competition between human groups in many different organizational levels are obvious in activities, e.g. of academic research teams, sports teams and armies. Although competition between groups is generally regarded as the ultimate selective force favouring costly within-group cooperation among non-related individuals (Wilson 1975; Avilés 2002; Boyd *et al.* 2003; West *et al.* 2007), it has received relatively little attention from empiricists in comparison with the various forms of reciprocity and punishment that have been suggested to function as proximate mechanisms allowing the maintenance of cooperation (Trivers 1971; Axelrod & Hamilton 1981; Nowak & Sigmund 1998; Wedekind & Milinski 2000; Fehr & Gächter 2002; Gürerk *et al.* 2006). The existing empirical studies, using various experimental designs, have shown that group competition promotes within-group cooperation (Erev *et al.* 1993; Gunnthorsdottir & Rapoport 2006; West *et al.* 2006; Tan & Bolle 2007). We studied cooperation in an experiment where reciprocation and communication among subjects was excluded, and where the effect of group competition was determined by the difference in the performance of the groups. This novel experimental set-up comes with a number of important benefits in comparison with earlier studies. First, it allows unravelling the effect of group competition in the absence of proximate mechanisms possibly maintaining cooperation. Second, it allows the derivation of the analytic solution for the relationship between the strength of group competition and expected level of cooperation. Third, and most important, it allows the assessment of the effect of group competition on total productivity in a straight forward manner, unlike the previous studies where group competition has been for a fixed external prize.

Human decision making is affected not only by rational calculations of material pay-offs, but also by emotions. Moral emotions originate from norms delineating socially acceptable behaviour. These norms are enforced by social control, but are also internalized so that violations of social norms elicit feelings of anger (when others violate norms) and guilt (when one violates norms oneself). It has been argued that the primary function of feelings of anger and guilt is to uphold cooperative relationships (Trivers 1971; Fessler & Haley 2003). In natural settings, deception elicits anger and moralistic aggression which serve as a credible threat of punishment, making deception less profitable. Guilt on the other hand makes deception less profitable by eliciting a feeling of internal discomfort, and also motivates reparative behaviour to mend damaged cooperative relationships. Quite revealingly, moral norms and emotions are usually highly group specific so that they primarily function to regulate within-group interactions, but are not applied to out-group members (Bernhard *et al.* 2006; Cohen *et al.* 2006). Internalized moral norms, and the resulting moral emotions, are likely to be the proximate reason explaining the outcome of certain experiments, e.g. the puzzling observations of ‘altruistic punishment’ in public goods games (Fehr & Gächter 2002; West *et al.* 2007). To gain insight into the emotions possibly affecting cooperative behaviour, we studied the effect of between-group competition on the perception of group members as competitors or collaborators, and on self-reported moral emotions: anger towards subjects who donate less and guilt when the subject earned more than other group members.

## 2. Material and methods

### (a) Experimental set-up

A total of 192 students (eight sessions with 24 subjects in each) took part in a decision-making experiment with real monetary stakes and two treatment conditions: public goods (PG) and public goods with group competition (GC). Game instructions given to subjects are available in the electronic supplementary material accompanying the paper. In the PG treatment, the subjects played the following game in groups of four (*n*=4): each subject received an endowment of 20 money units (MUs) and each one could contribute between 0 and 20 MUs to a group project. The subjects could keep the money that they did not contribute to the project. The number of MUs contributed to the group project by all the subjects was multiplied by the experimenter by factor *a* (*a*=2), and divided equally among the four group members. The benefit from investing one additional MU to the group project is

(2.1) $$B_{\text{PG}}=(-1)+\frac{a\times 1}{n}=(-1)+\frac{2\times 1}{4}=-\frac{1}{2}.$$

Thus, by investing one additional MU in the group project, a subject got a net benefit of −0.5 MUs, and it was in the material self-interest of any subject to keep all MUs privately—irrespective of how much the other three subjects contributed. Yet, if all the group members kept all MUs privately, each subject earned only 20 MUs, whereas if all of them invested their 20 MUs, each subject would earn (4×2×20)/4=40 MUs.

The GC treatment was otherwise identical with the PG treatment, except that after money in the group project was divided among the group members, a group competition effect was executed: two randomly drawn groups were paired and the difference in total MUs between the two groups was calculated. The group with more earnings then received *b* times (*b*=2) the difference in the total MUs between the groups, and the group with fewer earnings suffered the loss of an equal number of MUs. The winnings and losses were divided equally among the members of the respective groups. In the GC treatment, there is no single most profitable strategy, since profits gained from within-group competition are exactly cancelled by losses in the between-group competition. However, as in PG, investing in the group project always resulted in reduced income relative to other members of the group. Formally, the net benefit from investing one additional MU in the group project in the GC treatment was

(2.2) $$B_{\text{GC}}=\left((-1)+\frac{a\times 1}{n}\right)+\frac{b\times 1}{n}=\left((-1)+\frac{2\times 1}{4}\right)+\frac{2\times 1}{4}=0.$$

This means that no matter what a subject contributed to the group project, his/her private earnings were not affected. Investing more in the group project, however, increased the earnings of own-group members and decreased earnings of members of the competing group. As in the PG treatment, the collectively most profitable strategy would be for everyone to invest all MUs in the group project, because then all players earn 40 MUs, instead of 20 MUs when no one invests.

In both the treatments the investment decisions were anonymous. The subjects made their investment decisions simultaneously. After the decisions were made, the subjects were informed about the investments of the other group members and the resulting pay-offs. In the group competition effect, the players were informed of the total amount of MUs in their own and in the competing group, and the resulting effects to both groups and to their own-group members individually. All the subjects played 10 identical rounds of both games. Between rounds, the groups were randomly reconstructed so that the subjects never played the same game with the same people again. To examine the possible effects of playing one treatment before the other, half of the sessions consisted of 10 rounds of the PG treatment followed by 10 rounds of the GC treatment. In the other half of the sessions, the treatments were applied in reverse order. After playing both treatments, the subjects were asked to report their perception about group members in both treatments by ticking an appropriate position on a line spanning from collaborator to competitor. For both treatments, the subjects were also asked to report their feelings of anger towards own-group members who invested less than they did to the group project, and feelings of guilt when they earned more than the other subjects in the group. Emotions were reported by ticking an appropriate position on a line spanning from no emotion to strong emotion.

### (b) General experimental procedures

Subjects to the study were recruited from all faculties in the University of Jyväskylä with emails sent to student mailing lists, announcing a study involving playing a game on a computer and a chance to earn money. The subjects registered to attend a game session via the University course web service. The game sessions were held in two computer classrooms with 12 computers. Each computer was in a separate cubicle with a cloth covering the entrance. The subjects were directed to computers in a random order when 24 subjects had arrived. If there were more willing subjects than required, the last to arrive were given a 5 € show-up fee and excused. Subject names were not called out.

Once in the cubicle, the subjects first read a sheet of paper explaining that they were taking part in a decision-making experiment, that their earnings would depend on decisions made by themselves and the other subjects, and that all decisions and the amount of money earned would not be known by other subjects. The paper also included the sequence of events that would take place during the experiment. Next, the subjects read the instructions to the first game treatment (PG or GC, depending on the session), also on a sheet of paper (English translations of the original instructions in Finnish are given in the electronic supplementary material).

After everyone had read the instructions, the subjects were asked to put on earmuffs to exclude any auditory disturbance, and the experimenter started the computer software. The software first presented a series of questions to make sure that everyone understood the structure of the game. After all the subjects in the session had correctly answered all questions, the game started and ran automatically until the last round. After the last round, the subjects wrote on a sheet of paper the number of MUs they earned during the game (a backup for possible computer failure).

Next, the experimenter dealt out instructions for the second game (PG or GC, depending on the session). After reading the instructions, the subjects had to again answer a set of questions on a computer to make sure the instructions were understood. After all the subjects had correctly answered all the questions, the game started and ran automatically until the last round. After the last round of the second game, the subjects wrote down the number of MUs earned. The experimenter then handed out a questionnaire asking some background information about the subjects (age, sex, etc), and about emotions felt in the game. The subjects could also fill in a voluntary contact information sheet. Confidentiality of all the information was assured. After subjects had filled in the questionnaire, they were individually excused and paid in cash the amount of euros corresponding to their earnings in the whole game session.

### (c) Data and statistical analysis

Analysis of factors affecting investments in the group project is based on data, where the investments of all players in a session are averaged for each game round. Owing to a software problem, in one game there are investment data only for the first eight rounds, but this does not create any problems for data analysis.

We analysed the mean investment to the group projects by a linear mixed model procedure of SPSS statistical software (v. 14.0.2). We used the design of the basic cross-over model (table 1), where the full model includes the main fixed effects of the variables ‘period’, ‘order’, ‘round’, and all their interactions; and random effects of the variable ‘session’ (nested within order) and the interaction between ‘session’ and ‘period’. Variable ‘period’ codes for first and second game in a session. Variable ‘order’ codes for the order of the treatments (PG and GC) in a session. Variable ‘round’ codes for 10 rounds within the period. Variable ‘session’ codes for eight separate game sessions. By means of the cross-over model, we are able to control the carry-over effect between periods, as the investment during the latter period can be affected by the previous period. In the present model, the treatment effect (PG or GC) is equivalent to the period-by-order interaction.

Effects of game treatment on perception of group members and on emotions of anger and guilt were tested with paired samples *t*-test, comparing the values for each subject. For the analysis, the continuous perception data were coded so that collaborator got a value 0 and competitor value 100. Similarly, data for emotions were coded 0 for no emotion and 100 for strong emotion. Data for emotion of guilt were collected from seven sessions, data for perception of group members and on emotion of anger were collected for all eight sessions.

## 3. Results

The level of cooperation (i.e. the level of investments to the group project) was considerably higher in the GC treatment than in the PG treatment (figure 1; see table 1 for results of statistical tests where the period×order interaction corresponds to the treatment effect PG versus GC). As the overall earnings are a linear function of the level of cooperation, productivity measured as mean earnings over the 10 game rounds was also higher in the presence of group competition than in its absence (333 versus 251 MUs, paired samples *t*-test, *t*_167_=−21.98, *p*<0.001).

The order of the game treatments had a significant effect on the level of investments (order effect, table 1). Investments in the GC treatment were higher when the GC treatment was the second treatment than when it was the first treatment. This effect was possibly due to frustration from low earnings in the PG game, which then prompted higher investments in the following GC game. Game round affected investments differentially in the two treatments (significant treatment×round interaction, table 1). In the PG treatment, investments decreased as the game proceeded. This is a very general result in public goods games (Ledyard 1995). In the GC treatment, the average level of investments stayed nearly constant across rounds, possibly reflecting the independence of individual earnings of investment to group project and the consequent lack of a best single strategy for maximizing individual pay-offs.

Competition between groups affected the perception of group members and moral emotions in the game. Mirroring the increased level of cooperation in the GC treatment, the subjects perceived their group members more as collaborators in the GC treatment and more as competitors in the PG treatment (figure 2; paired samples *t*-test, *t*_190_=15.75 *p*<0.001). The self-reported feelings of anger towards free riders were fairly high in both treatments (figure 3), but significantly higher in GC treatment (mean increase 14.2 percentage points, paired samples *t*-test, *t*_191_=−6.25, *p*<0.001). The self-reported feelings of guilt from own uncooperative actions were generally quite low (figure 3), but also significantly higher in GC treatment than in the PG treatment (mean increase 4.9% points, paired samples *t*-test, *t*_164_=−2.88 *p*=0.004).

## 4. Discussion

Our study shows that between-group competition radically increases the level of within-group cooperation in the public goods social dilemma. The within-group social dilemma of the public goods game can be dissolved by between-group competition because between-group competition aligns individual and group interests (West *et al.* 2007), even in one-shot interactions where groups are reformed each round. Remarkably, the presence of between-group competition resulted in higher average earnings and increased overall productivity in our study. This result contrasts with studies on punishment as a mechanism promoting cooperation, because the costs of punishment usually outweigh the benefits of increased cooperation, leading to lower average earnings in games where punishment is allowed (Fehr & Gächter 2002; Egas & Riedl 2008; Herrmann *et al.* 2008; see also Gürerk *et al.* 2006).

A key factor in determining the importance of group competition for within-group cooperation is the relative strength of between- and within-group competition. In our study, the effect of between-group competition was only as strong as within-group competition, but it was sufficient to radically increase cooperation within groups. There can be little doubt that stronger between-group competition would result in even higher levels of within-group cooperation, as cooperation then would be the most profitable strategy for both individuals and groups. High levels of between-group competition can thus favour cooperation also among unrelated individuals. There are good reasons to believe that between-group competition is, and has been, severe in most human societies at least periodically. Human culture is full of legends of individuals joining forces in heroic self-sacrificial acts to defeat the common enemy. Records of war-like activity in chimpanzees (*Pan troglodytes*;Wilson & Wrangham 2003) and in pre-historic humans (Keeley 1996; Bowles 2006) suggest that between-group competition has been an important factor shaping human social behaviour during the evolutionary history of the species. In an intriguing recent study, Choi & Bowles (2007) showed that warfare may coevolve with the tendencies for altruism and out-group aggression. Thus, the level of between-group competition should not be assumed to be determined solely by forces external to the social system. Instead, group competition can be seen as both the engine and the legacy of the coevolutionary process (Arrow 2007).

In our experiment, subjects were anonymous and the groups were restructured every round. Thus, there were no possibilities for punishment or reparative behaviour, to which anger and guilt have been suggested to be functionally linked (Trivers 1971; Fessler & Haley 2003). These emotions, therefore, seem irrational in the context of both games. It seems likely that these emotions, which can result in personal benefits in real-life repeated interactions, cannot be voluntarily suppressed in anonymous, one-shot experimental settings (Richerson & Boyd 2005; West *et al.* 2007). Considering that the group-competition effect was executed via a computer terminal without any suggestion that the subjects should identify with members of their current group, the finding that levels of anger and guilt were elevated by group competition suggests the existence of emotional mechanisms promoting within-group cooperation in the face of group conflict. Further support for this hypothesis comes from studies where identification with group members has been found to increase the level of cooperation (De Cremer & Van Vugt 1999).

The public goods game with group competition is an illustrative framework for studying the consequences of between-group competition on within-group cooperation, but as all simplified models it has its limitations. In our experiment, interactions between groups were constrained to be zero-sum competitive interactions where wins by one group equalled losses of another group. In real life, group interactions can also benefit both groups, as in profitable trade, or be destructive to both groups, as in warfare without a clear winner. Group competition should thus not be seen as something that inevitably leads to higher productivity and pay-offs for members of a society. Rather, group competition should be seen as a force that has shaped human social behaviour during the evolutionary history, and a factual force shaping the daily lives of modern humans, from competition in working life to clashes between nations.

## Figures and Tables

**Figure 1:**
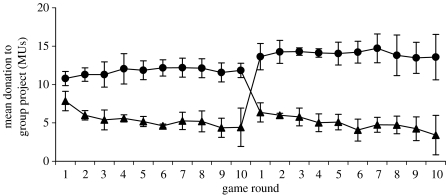
Investments to the group project. Triangles, PG treatment; circles, GC treatment. Symbols denote the mean of session means. Error bars denote 95% confidence interval of mean.

**Figure 2:**
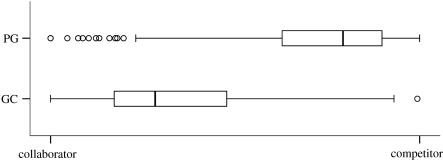
Box-plot figure of perceptions of own-group members on a scale from collaborator to competitor in the PG treatment and in the GC treatment.

**Figure 3:**
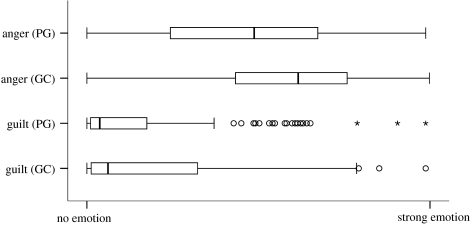
Box-plot figure of feelings of anger towards own-group members who donated less to the group project, and feelings of guilt when a subject earned more than own-group members. PG: public goods treatment; GC: public goods with group competition treatment.

**Table 1:** Results of mixed-model analysis on investments to the group project. (Variable ‘period’ codes for first and second game in a session. Variable ‘order’ codes for the order of the treatments (PG and GC) in a session. The interaction period×order tests for the effect of the treatment (PG versus GC) on investments to the group project. Session (order) and its interactions are random effects.)

	test statistic	*p*
intercept	*F*_1,6.024_=1416.21	0.000
period	*F*_1,6.019_=4.56	0.077
order	*F*_1,6.024_=7.88	0.031
round	*F*_9,106.082_=1.75	0.086
treatment (=period×order)	*F*_1,6.019_=6.02	0.000
order×round	*F*_9,106.082_=0.32	0.969
period×round	*F*_9,106.082_=0.43	0.914
treatment×round (=period×order×round)	*F*_9,106.082_=3.36	0.001
session (order)	*Z*=0.22	0.825
period×session (order)	*Z*=1.41	0.159

## Supplementary Material

Game instructions Game instructions for ‘Between-group competition and human cooperation’
