# Supplementary material for: Between-group competition and human cooperation
Source: Proc Biol Sci. 2008 Sep 30;276(1655):355–60. doi: 10.1098/rspb.2008.1060 (PMC2581672; doi:10.1098/rspb.2008.1060)
Supplement: Supplementary file 1 [file rspb20081060s18.doc]

Supplementary Material for

**Between-group competition and human cooperation**

M. Puurtinen and T. Mappes

Game instructions

**Instructions for PG treatment**

**Game instructions**

All players get these same instructions.

The game consists of 10 rounds. In each round you will have to make similar decisions.

In the beginning of a round the players are divided to groups of four players. **Group members are anonymous**, so you will not know who the other members are, and the other members will not know who you are.

**After each round the players are randomly divided to new groups.** The players in your group thus change after each round, and you never play two rounds with the same players. The players in your group are referred to with the color of their tokens (yellow, green, and blue). You will always play with red tokens.

In the game you make decisions about allocation of tokens. **Value of one token is 2 cents** (0,02 €).

**Progress of the game:**

1. In the beginning of a round you are given 20 tokens.
2. Next you decide how many tokens (0-20) you invest to common group project and how many tokens you keep for yourself. The other three members of the group make their decision about the use of their tokens simultaneously.
3. After all group members have made their decision, the number of tokens invested to the group project is summed, and game organizer doubles the amount of tokens in the project.
4. The doubled amount of tokens is divided equally among the group members.

**Your tokens after a round**:

Tokens you keep for yourself

*plus*	tokens you earn from the project.

**Group project – example 1**

All 4 players invest 20 tokens to the project, 80 tokens in total.

The number of tokens in the project is doubled to 160 (2×80) tokens.

The tokens from the project are divided equally among players, 40 tokens for each.

All players earned 40 tokens.

**Group project – example 2**

Three players (A, B, and C) invest 20 tokens to the project; one player (D) invests 0 tokens. In total 60 tokens were invested to the project.

The number of tokens in the project is doubled to 120 (2×60) tokens.

The tokens from the project are divided equally among players, 30 tokens for each.

Players A, B, and C earned 30 tokens, player D earned 50 tokens (20 tokens D kept for herself *plus* 30 tokens from the group project).

5. The number of tokens you earned in the round is stored in the computer memory. These tokens cannot be used in following rounds. The number of tokens you earned is shown in the end of each round. In the beginning of new round you are given another 20 tokens to play with.

**Instructions for GC treatment:**

**Game instructions**

All players get these same instructions.

The game consists of 10 rounds. In each round you will have to make similar decisions.

In the beginning of a round the players are divided to groups of four players. **Group members are anonymous**, so you will not know who the other members are, and the other members will not know who you are.

**After each round the players are randomly divided to new groups.** The players in your group thus change after each round, and you never play two rounds with the same players. The players in your group are referred to with the color of their tokens (yellow, green, and blue). You will always play with red tokens.

In the game you make decisions about allocation of tokens. **Value of one token is 2 cents** (0,02 €).

**Progress of the game:**

1. In the beginning of a round you are given 20 tokens.
2. Next you decide how many tokens (0-20) you invest to common group project and how many tokens you keep for yourself. The other three members of the group make their decision about the use of their tokens simultaneously.
3. After all group members have made their decision, the number of tokens invested to the group project is summed, and game organizer doubles the amount of tokens in the project.
4. The doubled amount of tokens is divided equally among the group members.

**Your tokens after the group project**:

Tokens you keep for yourself

*plus*	tokens you earn from the project.

**Group project – example 1**

All 4 players invest 20 tokens to the project, 80 tokens in total.

The number of tokens in the project is doubled to 160 (2×80) tokens.

The tokens from the project are divided equally among players, 40 tokens for each.

All four players have 40 tokens.

**Group project – example 2**

Three players (A, B, and C) invest 20 tokens to the project; one player (D) invests 0 tokens. In total 60 tokens were invested to the project.

The number of tokens in the project is doubled to 120 (2×60) tokens.

The tokens from the project are divided equally among players, 30 tokens for each.

Players A, B, and C have 30 tokens, player D has 50 tokens (20 tokens D kept for herself *plus* 30 tokens from the group project).

5. Next, a comparison is made between the value of your table (the total sum of tokens in the group) and the value of another, randomly selected table. If the value of your group’s table exceeds the value of the other group’s table, wins your group twice the difference in the values of the tables. Correspondingly, if the value of your group’s table is lower than the value of the other group’s table, loses your group twice the difference in the values of the tables.
6. The wins or losses from group comparison are divided equally* among members of the group. The players account for group losses with all tokens from the current round (tokens earned from the group project and tokens not invested to the group project).

^*^ In case some group member of a group does not have enough tokens to cover her share of the losses, other members of the group will account for the proportion of losses the player cannot account for. If the total loss for the group exceeds the total amount of tokens in the group, the group loses all tokens from the current round. However, negative earnings will not be dealt to anyone. The winning group can only win as many tokens as the losing group has in the current round.

**Your tokens after a round:**

Tokens after the group project

*plus/minus* $\frac{2\times\text{difference in table values}}{4}$

**Group comparison – example**

The value of your group’s table is 140 tokens, and the value of the table being compared to is 120 tokens. The difference in table values is
140-120 = 20 tokens. Because the value of your group’s table is larger, you get 2×20 = 40 tokens from the other group. This sum is divided equally among the members in your group: 40÷4 = 10 tokens for each.

Correspondingly, members of the other group lose 10 tokens each.

7. The number of tokens you earned in the round is stored in the computer memory. These tokens cannot be used in following rounds. The number of tokens you earned is shown in the end of each round. In the beginning of new round you are given another 20 tokens to play with.
